# Supplementary material for: Degenerated Virulence and Irregular Development of Fusarium oxysporum f. sp. niveum Induced by Successive Subculture
Source: J Fungi (Basel). 2020 Dec 21;6(4):382. doi: 10.3390/jof6040382 (PMC7767292; doi:10.3390/jof6040382)
Supplement: Supplementary file 1 [file jof-06-00382-s001.pdf]

Supplementary Materials:

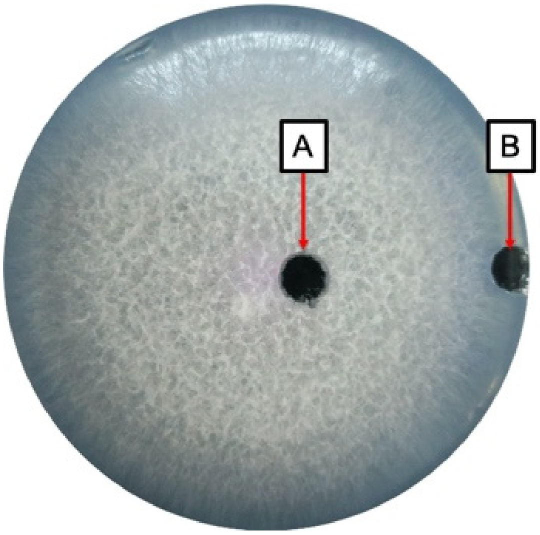

Supplementary Figure S1. Scheme of Fon successive cultural sources which are aged mycelium (A) and hyphal tip (B).

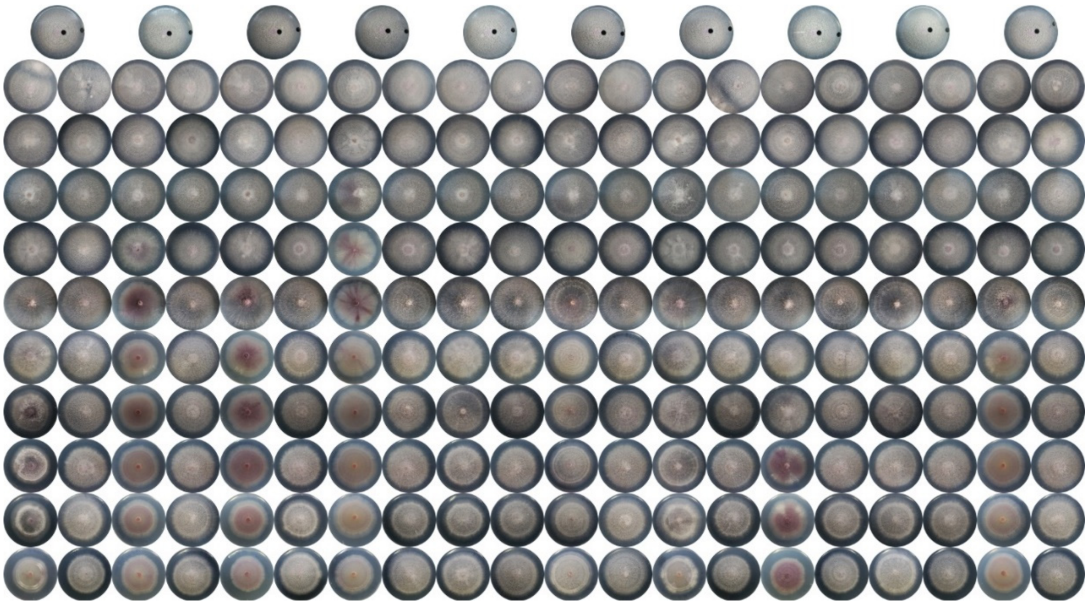

Supplementary Figure S2. Snapshots of Fon successive culture phenotype. Fon were grown on half-strength PDA and successive cultured for 10 generations. Each successive subculture was aligned in two lanes. Every two lanes indicated cultures from one origin where cultural discs were from aged mycelium (left lane) and hyphal tip (right lane).

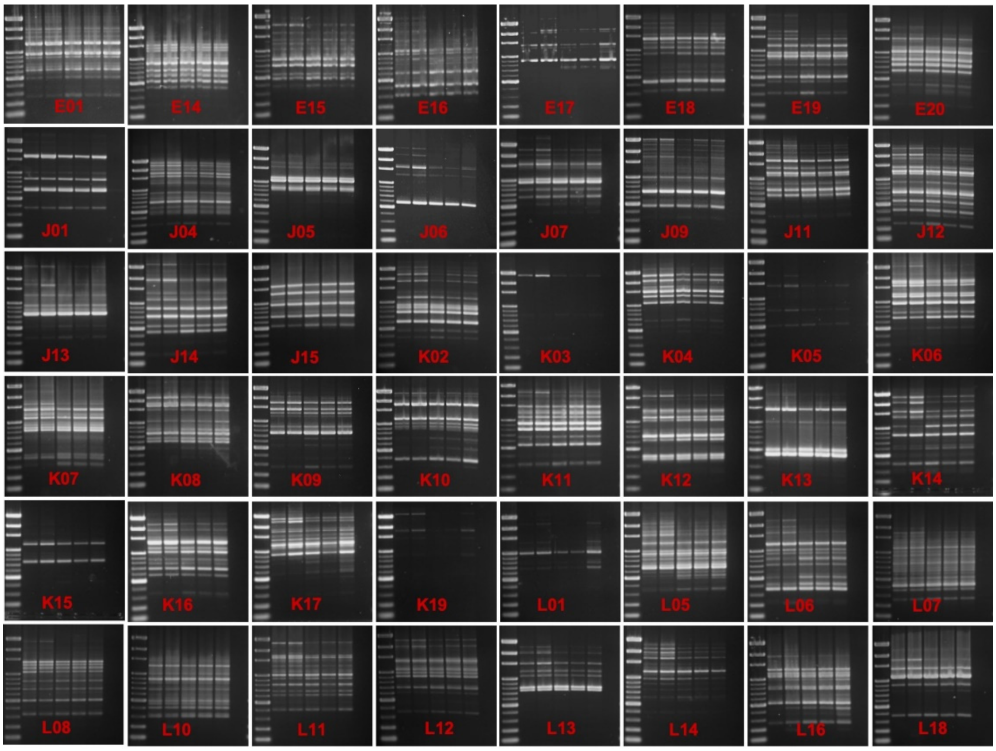

Supplementary Figure S3. The random amplified polymorphic DNA (RAPD) patterns of different *F. oxysporum* f. sp. *niveum* (Fon-H0103) variants. Lanes of each gel figures from left to right are DNA molecular marker (100 bp marker, IDBio Ltd, Taiwan), Fon variants PT2, PT3, PT11, and MT6, and Fon ST, respectively. Total 48 random primers (Operon Technologies Inc., Alameda, CA, USA) were used and indicated in figures.
